# MS Prevalence and Patients' Characteristics in the District of Braga, Portugal

**DOI:** 10.1155/2015/895163

**Published:** 2015-01-08

**Authors:** José Figueiredo, Ângela Silva, João J. Cerqueira, Joaquim Fonseca, Paulo A. Pereira

**Affiliations:** ^1^Neurology Department, Hospital Privado de Braga, 4715-196 Braga, Portugal; ^2^Neurology Department, Hospital Senhora da Oliveira de Guimarães, 4835-044 Guimarães, Portugal; ^3^School of Health Sciences, Universidade do Minho, 4710-057 Braga, Portugal; ^4^Neurology Department, Hospital de Braga, 4710-243 Braga, Portugal; ^5^Novartis Farma SA, 2740-255 Porto Salvo, Portugal; ^6^Department of Mathematics and Applications, Universidade do Minho, 4800-058 Guimarães, Portugal

## Abstract

Multiple Sclerosis (MS) is a chronic autoimmune disease of the Central Nervous System causing inflammation and neurodegeneration. There are only 3 epidemiological studies in Portugal, 2 in the Centre and 1 in the North, and there is the need to further study MS epidemiology in this country. The objective of this work is to contribute to the MS epidemiological knowledge in Portugal, describing the patients' epidemiological, demographic, and clinical characteristics in the Braga district of Portugal. This is a cross-sectional study of 345 patients followed in two hospitals of Braga district. These hospitals cover a resident population of 866,012 inhabitants. The data was collected from the clinical records, and 31/12/2009 was established as the prevalence day. For all MS patients, demographic characteristics and clinical outcomes are reported. We have found an incidence of 2.74/100,000 and a prevalence of 39.82/100,000 inhabitants. Most patients have an EDSS of 3 or lower and a mean age of 42 years. The diagnosis was done at mean age of 35, with RRMS being the disease type in more than 80% of patients. In this cohort, we found a female : male ratio of 1.79. More than 50% of patients are treated with Interferon *β*-1b IM or IFN*β*-1a SC 22 *μ*g.

## 1. Introduction

Multiple Sclerosis (MS) is a chronic autoimmune disease of the Central Nervous System (CNS) causing inflammation and neurodegeneration. The characteristic demyelination of the neurons, followed in many cases by axonal loss and gliosis, results in incapacity progression [1]. The incapacity status is usually assessed in MS patients with the Expanded Disability Status Scale (EDSS) [2]. Different types of MS have been described based on the clinical course: Relapsing-Remitting Multiple Sclerosis (RRMS), Secondary Progressive Multiple Sclerosis (SPMS), Primary Progressive Multiple Sclerosis (PPMS), Relapsing-Progressing Multiple Sclerosis (RPMS) [3] and Clinically Isolated Syndrome (CIS) [4].

There is yet no cure for MS, but several disease modifying treatments have shown to have beneficial effects on the disease progression. The approved treatments for MS in Europe include Interferon *β*-1a SC and IM, Interferon *β*-1b SC, Glatiramer Acetate SC, Natalizumab IV, oral Fingolimod, oral Dimethyl Fumarate, oral Teriflunomide, and Alemtuzumab IV.

MS has a great social and economic impact, because it affects mainly young adults [5], who will be more prone to unemployment [6] and in a big percentage will need a walking aid few years after the disease onset [7, 8]. MS is more prevalent in females [9] and Caucasians [10] and it is influenced by the geographic localization (Multiple Sclerosis International Federation).

Our aim is to contribute to the MS epidemiological knowledge in Portugal, describing the patients' epidemiological, demographic, and clinical characteristics.

## 2. Methods

The district of Braga is located in the Northwestern part of Portugal. In the district of Braga, there are two hospitals with consultation and treatment of multiple sclerosis for a resident population of 866,012 inhabitants (INE). The hospitals are Hospital de São Marcos in the city of Braga (HSM) and Hospital Senhora da Oliveira in the city of Guimarães (HSO). Braga is the youngest district of Portugal: 30.7% of the population is under 25 years, 56.4% are between 25 and 64 years, and 12.9% have more than 64 years. The population of this district is mostly lower middle class (which covers 37.1% of the population).

This is a cross-sectional study of MS patients followed in the Braga and Guimarães Hospitals. The data was collected from the clinical records, and 31/12/2009 was established as the prevalence day. At the prevalence date, only Interferon *β*-1a SC and IM, Interferon *β*-1b SC, Glatiramer Acetate SC, and Natalizumab IV were approved for MS in Europe. All the cases were assessed by a neurologist and included in the study whenever they met the Poser diagnostic criteria. Only residents of Braga district which had an office visit between the 1st of January 1997 and the 31st of December 2009 were included in this study. We requested the necessary consent to patients, Portuguese Data Privacy Committee (Comissão Nacional de Proteção de Dados), and to the Ethical Committees of both hospitals. There were no clinical records for HSM patients before 1997. In order to compare the hospitals, the year of diagnosis for HSO patients was considered to be 1997 for every patient that had records older than that.

In this study, for all MS patients, demographic characteristics (sex, age, age at diagnosis, treatment, and spatial distribution of place of residence) and clinical outcome (type of the disease, treatment, clinical severity score [EDSS], and year and month of diagnosis) are reported. Student's *t*-test and Chi-squared test were used to evaluate differences between groups. All significance tests were based on *P* < 0.05 as the level of significance.

## 3. Results

The database is composed of 345 individuals that are followed in the MS outpatient clinic of HSM and HSO. The estimated disease prevalence is 39.84 per 100,000 inhabitants (95% CI = 27.47–52.21). The female : male ratio is 1.79, with 64.06% of females MS patients and 35.94% males.

The mean age at diagnosis is approximately 35 years and varies between 13.61 and 70.55 years (Table 1). At prevalence day, the mean age is 42.

HSM and HSO have a similar distribution of patients over the RRMS and SPMS forms. The number of patients with PPMS and CIS forms is too low be compared between hospitals (Table 2).

**Figure 1:**
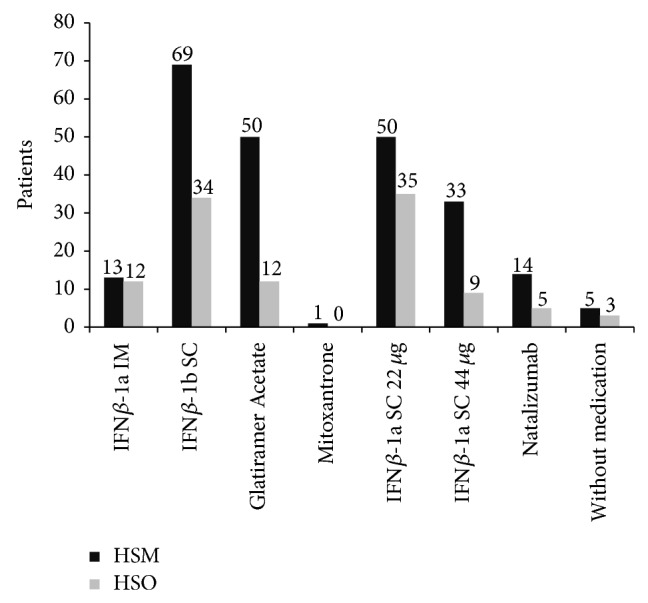
Most patients are treated with first-line drugs.

The most common treatment is Interferon *β*-1b IM, with 29.86%, followed by IFN*β*-1a SC 22 *μ*g with 24.64%, Glatiramer Acetate with 17.97%, IFN*β*-1a SC 44 *μ*g with 12.17%, IFN*β*-1a IM with 7.25%, Natalizumab IV with 5.51%, and Mitoxantrone 0.29%. 2.32% of patients are without treatment (Figure 1).

**Figure 2:**
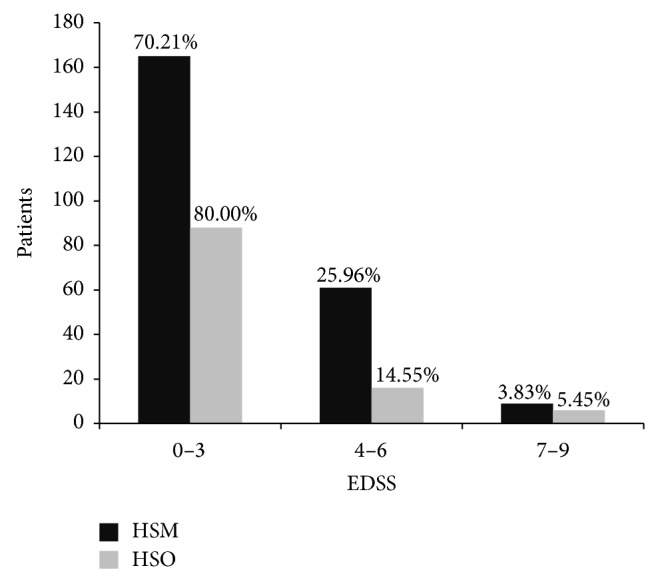
The patient distribution in these three EDSS groups is similar in both hospitals (*P* = 0.0549).

Patients were divided in three groups according to their disability as quantified by the EDSS. Most patients have an EDSS of 3 or less (Figure 2).

**Figure 3:**
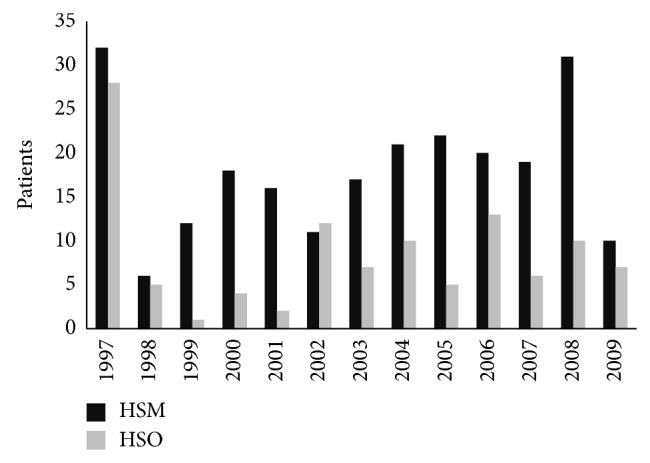
The annual incidence between 1998 and 2009 was 2.74/100,000.

In HSM, there were no MS patient records before 1997. HSO MS patients have records for previous years, but we decided to consider 1997 as the year of diagnosis in those cases. If we assume 866,012 as the Braga district population from 1998 to 2009, we can estimate an average annual incidence of 2.74/100,000 inhabitants in the Braga district (Figure 3).

## 4. Discussion

MS patients in Portugal are almost exclusively followed in public hospitals because Disease Modifying Treatments (DMTs) are almost only offered to patients without any copayment in the National Health System (NHS) hospitals. As a result, only a very small number of patients, at national level, have access to DMTs outside the NHS hospitals. HSM and HSO are the only MS reference centers in the district of Braga, so it is expected that the vast majority of MS patients of this district be registered in one of these two hospitals. There is the possibility that some MS patients from this district are followed in other MS centers in Portugal, so the estimated prevalence may be underestimated. To more accurately determine the MS prevalence in this district, further work should be done. A cross-check between the hospitals and the primary care centers patient's lists would give the most accurate prevalence numbers. Additionally, MS patient's lists, from hospitals like Centro Hospitalar de São João, Centro Hospitalar do Porto, and Centro Hospitalar e Universitário de Coimbra should be checked to capture patients with residence in Braga district, who are followed in those hospitals.

For several years, Portugal has been considered to be a low-medium prevalence zone for MS [11], but recent epidemiological studies suggest that our country should instead be considered a medium prevalence zone [12, 13]. A prevalence study in the district of Santarém [13] determined a prevalence of 46.3 cases/100,000 in 2006 and a nationwide survey presented in 2011 by J. Pinheiro found this number to be 54/100,000 [14]. More recently, a prevalence study in a referral hospital and three community health clinics in a large city (Lisboa) determined a prevalence of 56.2/100,000 [15]. The 39.84/100,000 prevalence that we estimated in this study is probably underestimated due to the exclusion of patients who are resident in Braga district but are followed in other hospitals from cities like Oporto or Coimbra. The 2.74/100,000 annual incidence is lower than that reported for Spain and Italy [16], but this value is probably underestimated for the same reason and also because we considered a constant population (equal to 2009 numbers) between 1998 and 2009.

The gender ratio F : M in this study is 1.79. In both hospitals, the highest number of diagnosis was in the year 2008 (excluding 1997) with 11.88% of the patients. The MS diagnostic of HSO patients was made at a younger age. The age at diagnosis, patient distribution amongst MS forms, and disability status assessed by EDSS is also consistent with the results from previous MS studies [7, 16]. The low percentage of patients without treatment, 2.32%, is noteworthy.

## 5. Conclusion

The district of Braga is a medium prevalence zone for MS with a prevalence of 39.82/100,000 inhabitants. We estimated an average annual incidence of 2.74/100,000 inhabitants between 1998 and 2009. In this study, MS patients' demographic and clinical characteristics are consistent with what was expected in light of current scientific literature.

## Figures and Tables

**Table 1:** Age at diagnosis.

	Minimum	Maximum	Mean	Median	Std. deviation	1st Q	3rd Q
Total	**13,61**	**70,55**	**35,38**	**34,91**	**11,58**	**26,09**	**42,24**
HSM	14,82	70,55	36,09	35,84	11,75	26,98	44,41
HSO	13,61	65,83	33,84	33,22	11,10	25,72	39,85

The mean age at diagnosis is 35.

**Table 2:** Most of patients have the Relapsing-Remitting form of Multiple Sclerosis.

MS form	HSM		HSO		Total	
	*n*	%	*n*	%	*n*	%
CIS	2	0,85%	1	0,91%	3	0,87%
RRMS	190	80,85%	96	87,27%	286	82,90%
SPMS	37	15,74%	12	10,91%	49	14,20%
PPMS	6	2,55%	1	0,91%	7	2,03%
Total	**235**		**110**		**345**	

## Abbreviations

MS: Multiple Sclerosis
CNS: Central Nervous System
EDSS: Expanded Disability Status Scale
RRMS: Relapsing-Remitting Multiple Sclerosis
SPMS: Secondary Progressive Multiple Sclerosis
PPMS: Primary Progressive Multiple Sclerosis
RPMS: Relapsing-Progressing Multiple Sclerosis
CIS: Clinically Isolated Syndrome
HSM: Hospital de São Marcos
HSO: Hospital Senhora da Oliveira
DMTs: Disease Modifying Treatments
NHS: National Health System.

## Highlights

(i) 345 MS patients followed in Braga district and (ii) patient's demographic characteristics (sex, age, age at diagnosis, treatment, and spatial distribution of place of residence) and clinical outcome (type of the disease, treatment, clinical severity score [EDSS], and year and month of diagnosis) were collected.
